# Anxiety, Difficulties, and Coping of Infertile Women

**DOI:** 10.3390/healthcare9040466

**Published:** 2021-04-15

**Authors:** Diana Antonia Iordăchescu, Corina Ioana Paica, Alina Estera Boca, Corina Gică, Anca Maria Panaitescu, Gheorghe Peltecu, Alina Veduță, Nicolae Gică

**Affiliations:** 1 Faculty of Psychology and Educational Sciences, University of Bucharest, 90, Panduri Avenue, 050653 Bucharest, Romania; diana.antonia.vesman@drd.unibuc.ro (D.A.I.); corina.paica@unibuc.ro (C.I.P.); alina.boca19@gmail.com (A.E.B.); 2 Filantropia Clinical Hospital, 011132 Bucharest, Romania; anca.panaitescu@umfcd.ro (A.M.P.); gheorghe.peltecu@umfcd.ro (G.P.); alina.veduta@gmail.com (A.V.); gica.nicolae@umfcd.ro (N.G.); 3 Department of Obstetrics and Gynecology, Carol Davila University of Medicine and Pharmacy, 050474 Bucharest, Romania

**Keywords:** infertility, anxiety, coping strategies, difficulties, women

## Abstract

The present study aims to highlight how women perceive and adapt to infertility difficulties. To better understand the difficulties that women diagnosed with infertility are experiencing, the study explores this concept in correlation with anxiety and coping. 240 women with fertility problems from various parts of Romania completed the State-Trait Anxiety Inventory (STAI-Form Y), Brief COPE and the scale “Difficulties With Infertility and Its Treatment.” Statistical analyzes showed that women who were at the beginning of treatment obtained higher scores on the anxiety [F(2,237) = 4.76, *p* = 0.009] and on the difficulties scale [F(2,237) = 3.53, *p* = 0.031], compared to participants who resorted to repeated fertilization procedures. It is important to emphasize that there is a significant positive correlation between the perception of infertility difficulties and coping, and also between difficulties and state anxiety. Regarding the relationship between state anxiety and coping, there were significant positive associations between maladaptive coping strategies and state anxiety, while adaptive strategies were negatively associated with state anxiety. In addition, regarding coping strategies, venting and self-blame occurred predominantly in women who know that the cause of infertility is female-related. These findings draws attention to the fact that infertile women live this experience at very high levels of anxiety, using quite a few adaptive coping mechanisms. These results highlight the need to investigate ways to reduce anxiety and optimizing adaptive coping strategies.

## 1. Introduction

Infertility is defined by the World Health Organization as the inability to conceive after one year or more in couples with regular sexual activity [1]. Infertility is an important public health issue that affects 1 of every 4 couples in Romania, according to the Association for Human Reproduction from Romania 2018 report [2].

Among the emotional aspects of women facing infertility, uncertainty and anxiety are significant issues. Women have higher rates of anxiety levels associated with infertility [3] and report infertility as one of the most stressful experiences in their life [4].

Anxiety is an adaptive natural response of the body to stressful events, and anxiety disorders are the most common mental issue in infertility, having a similar prevalence across different cultures [5].

While some studies show that there is a relationship between the duration of infertility, psychological factors (such as difficulties associated with infertility), and anxiety [6,7,8], others show that women who have a medium or long duration of infertility have low values of state anxiety [9,10].

Some studies show that anxiety remains high at all stages of the treatment cycle [11] or may be higher in patients who are on their first IVF treatment [12]. Other studies show that challenging and complicated repeated fertilization procedures represent a significant emotional difficulty for women, resulting in anxiety [13,14,15]. One possible explanation is that women may be emotionally exhausted by repeated fertilization procedures and have a significant decrease in stress coping strategies compared to women who are at the beginning of treatment [11]. Another possible explanation may be the three-month waiting time between two treatment cycles [16].

Women are the main patients when a couple experiences fertility issues, regardless of the cause of infertility, that being the reason why women are the focus of this study [17]. However, determining the cause of infertility is not always precise and clear. It can be a female cause (such as ovarian or gynecological conditions, polycystic ovary syndrome, or endometriosis), male cause (such as low sperm production, abnormal sperm function), both, or the diagnosis of unexplained infertility. Knowing the root cause of infertility can reduce the burden for women [18] because they understand better the diagnosis, while women with unexplained infertility do not know why they cannot get pregnant and become obsessed with finding an explanation [19,20].

Some studies show that there are no differences regarding anxiety depending on the cause of infertility in the women evaluated [7,21,22]. A recent study [23] found significantly higher levels of anxiety in women whose cause of infertility was female. The same researchers point out that when the cause of infertility is exclusively female, women experience higher levels of anxiety both before and during treatment, which may be associated with a sense of guilt. In one study [12], only 18% of infertile women had male factors as the cause of their infertility. This is in consonance with previous works, which argued that since women are ultimately the ones to conceive and become pregnant, infertility is often regarded as a woman’s problem whether or not the cause has been determined to be male factor infertility. In addition, Benyamini [24] shows that judging from a social view, women coping with infertility is related to the importance they place on parenthood and to actively undergoing fertility treatments.

The consequences of emotional burden in women dealing with infertility is not well understood and not often addressed in the literature. Benyamini et al. [25] focused on the factors that influence the level of stress during infertility treatment; they described five relevant key factors: Uncertainty and lack of control, family and social pressures, impact on self and spouse, treatment-induced problems, and treatment-related procedures. The impact on self and spouse refers to the lack of spontaneity in the sexual relationship, the worry that the medical treatment will cause physical harm in the long run, the impact of the fertility problems on the way women see themselves, and the impact of the fertility disorders on the way partners see themselves. Uncertainty and lack of control refer to the monthly anticipation of treatment results, the uncertainty regarding the future, and the feeling of lack of control over their life. Family and social pressures refer to the questions about childbearing. A recent meta-analysis based on studies from the US and Europe also showed that a blocked parenthood goal can lead to greater anxiety yet not necessarily to disengagement from the goal, and even when women disengage, it does not reduce their anxiety [26].

Treatment-induced disorders refer to the pain and physical discomfort of the couples. The economic aspects related to the treatment and treatment-related procedures refer to the bureaucratic procedures accompanying medical services, the disruption of functioning at work, and the relationship with the medical staff [25].

In order to overcome a stressful situation, people use a variety of coping styles. Problem and emotional focused strategies are two main coping strategies described in various studies [27]. Emotion-focused strategies have the purpose of regulating negative emotions and diminishing stress; examples of emotion-focused strategies include escaping and avoidance. In contrast, problem-focused strategies involve dealing directly with stressful situations, seeking support and information.

Many studies focus on adaptive versus maladaptive coping strategies. Consequently, problem-focused coping is considered an adaptive coping style, whereas avoidant coping is deemed maladaptive—being associated with anxiety and, in some cases, with negative health behaviors that can also affect reproduction (smoking, drinking alcohol and taking drugs, poor sleep, weight issues) [28].

Infertility is a source of stress that can impact the wellbeing of people with fertility disorders. This impact is influenced by the coping strategies: Active-confronting (asking for advice), active-avoidance (avoiding contact with pregnant women, keeping feelings for themselves), passive-avoidance (expecting a miracle, waiting as the only solution), meaning-based coping (linking the experience with the improvement of the marriage or personal growth) [29].

Studies that involved women undergoing in vitro fertility (IVF) treatment showed that those that mainly used emotion-focused strategies had higher levels of stress and more difficulties adjusting to their situation. Furthermore, denial as part of an avoidant coping strategy predicted higher stress levels related to infertility [30].

The results of a recent study [31] showed a positive association between seeking social support, avoidance coping, and state anxiety—these findings support the results of [32].

Positive attitude coping was negatively associated with State Anxiety, at least regarding women [31], similar to the results of Benyamini [17], which studied coping strategies centered on positive reframing.

The results of another study [33] discovered that passive coping (meaning emotion-focused coping) was a positive predictor for stress, while active coping (problem-focused coping) was a negative predictor for stress. Regarding emotion-focused and problem-focused strategies, the difference between couples was not significant, but women had less self-control compared to men, according to Yazdani et al. [34]. In addition, anxiety associated with infertility had a direct influence on active-oriented coping strategies.

Taking into account a few of the difficulties that women with infertility face (among them being uncertainty and lack of control), Gourounti et al. [35] examined the association between perception of infertility controllability and coping strategies, the results demonstrating a positive association between the perception of low controllability and avoidance coping. In addition, there was a positive association between the perception of a high level of controllability and problem-focused coping.

The important aim of infertility research is to improve clinical practice and optimize the chances of people with fertility problems achieving parenthood. Thus, researchers have to address questions that are pertinent to people with infertility, use appropriate methods, and report results in a comprehensive, transparent, and accessible manner.

In the desire to better understand the difficulties that women diagnosed with infertility are experiencing, the study explores this concept in correlation with anxiety and coping. Therefore, our specific aims and research hypotheses are:

**Hypothesis** **1 (H1).** *To investigate the relationship between infertility duration and anxiety level in infertile women. We hypothesized that women who experience an increased duration of infertility are associated with a higher level of anxiety and difficulties.*

**Hypothesis** **2 (H2).** *To assess if women who have repeated fertilization procedures have higher scores on anxiety and difficulties scales compared to those who have not started treatment yet or are at the beginning of it. We hypothesized that women who resort to repeated fertilization procedures have higher scores on anxiety and difficulties scales than those who have not yet started treatment or are at the beginning of it.*

**Hypothesis** **3 (H3).** *To explore if there is an association between anxiety, perception of difficulties, and coping. We hypothesized that there is a positive association between the perception of infertility difficulties and coping strategies in infertile women.*

**Hypothesis** **4 (H4).** *We also hypothesized that there is a positive association between the perception of infertility difficulties and state anxiety.*

**Hypothesis** **5 (H5).** *Likewise, we hypothesized that there is a positive association between state anxiety and maladaptive coping strategies and a negative association between state anxiety and adaptive coping strategies.*

**Hypothesis** **6 (H6).** *To investigate that there are differences between participants regarding difficulties, anxiety, and coping strategies depending on the cause of infertility (female, male, mixed, or unexplained). We hypothesized that there are differences between participants regarding difficulties, anxiety, and coping strategies depending on the cause of infertility (female, male, both, or unexplained).*

## 2. Materials and Methods

### 2.1. Design

The current study was designed as a non-experimental, descriptive, correlational study in which various hypotheses were tested.

### 2.2. Procedures and Participants

The present study was conducted between October and December 2019 and was approved by the Research Ethics Committee of the University of Bucharest. The participants were recruited from several Romanian cities through announcements posted on social media, inviting them to take part in a series of scientific studies whose main objective was the psychological evaluation and subsequently, the development of a counseling program, with the purpose of reducing anxiety and optimizing coping strategies.

Data were collected online through the Google Form platform. Before completing the questionnaires, the participants received information regarding the purpose of the study, data collection, and storage methods. The participants took part in this research voluntarily and expressed their agreement to participate in the study.

In addition, the research ethics principles were respected: The confidentiality of data and anonymity of the participants. The instruments used and work procedure were noninvasive and did not involve the participants in stressful or frustrating situations.

### 2.3. Instruments

Participants completed a set of tests that included: Socio-demographic information (age, marital status, education level-these represent the study covariables), information about infertility (duration, cause, number of fertilization’s) that represent the independent variable (duration was coded with 1, 2, or 3, according to Mahnaz Ashrafi [36]: (1) 1–2 years; (2) 2–5 years; (3) more than 5 years). Participants also completed the following psychological scales (these represent the dependent variables of the study):

(a) For the assessment of anxiety, we used the 20 items of the State Scale of State-Trait Anxiety Inventory (STAI-S) [37]. The questionnaire is the most used instrument for measuring anxiety. The S-Anxiety Scale reflects a transitory emotional state or a condition [17] that is characterized by subjective feelings of tension, distrust, nervousness, and worry. Items are rated on a 4-point Likert scale, from 1 (Not at all-no anxiety) to 4 (Very much-high anxiety), thus the range of total scores that can be obtained is from 20 to 80 (higher scores indicates higher anxiety). Ten items required reverse scoring. Examples of items: “I feel calm.”; “I am tense.” For Romania, the fidelity indices were between 0.85 and 0.95. For this study, the Cronbach alpha internal consistency coefficient was 0.94.
(b) To evaluate the difficulties of women diagnosed with infertility, we used the instrument “Difficulties with Infertility and Its Treatment” [25]. The questionnaire included 22 items divided into 5 subscales, scored on a 5-point Likert scale from 1 “not significant” to 5 “highly significant”. Examples of items: “I experience difficulty with the monthly anticipation of treatment results/ Questions and family pressure about childbearing/Lack of support from my partner/Pain and physical discomfort involved in treatment/The relationship with the medical staff.” The Cronbach alpha fidelity coefficients reported by the author were between 0.60–0.82. For the present study, the fidelity coefficients were: α = 0.90 (for the whole questionnaire), and for each subscale: 0.85 (“Uncertainty and lack of control” subscale), 0.82 (“Family and social pressures”), 0.70 (“Impact on self and spouse”), 0.62 (“Procedures related to treatment”), 0.44 (“Problems induced by treatment”). Due to the low value of internal consistency, this last subscale was not included in statistical analysis.
(c) The tool “Brief COPE” [38] was used to identify coping strategies. The instrument had 14 subscales with 2 items each. Each of these subscales was broadly classified into 2 major types of coping that have been identified in the literature: Problem-focused coping and emotion-focused coping. Active coping, use of instrumental support, planning, and acceptance were considered problem-focused coping, while self-distraction, denial, substance use, use of emotional support, behavioral disengagement, venting, positive reframing, humor, religion, and self-blame were considered emotion-focused coping [39]. The Cronbach’s alpha coefficient for this study varied for each subscale from 0.55 to 0.94.

## 3. Results

### 3.1. Sample Characteristics

Table 1 describes the demographic and fertility characteristics of infertile women. The research sample consisted of 240 women with fertility problems (*N* = 240). Women were aged between 22 and 46 years old (M = 32.71, SD = 4.85). As concerning education, most of them had bachelor’s degrees (43.8%), and less of them just finished elementary school (2.9%) or did not obtain a bachelor’s degree (2.9%). Most of them were married (88.8%).

Regarding duration, type, and cause of infertility, most of them presented an average duration of infertility, between 2–5 years (43.3%), primary infertility (65.8%), and the most common cause was female factor infertility (36.7%). Most of the women either had not yet begun treatment or were on multiple attempts of fertilization.

### 3.2. Statistical Analyses

Microsoft Excel and IBM SPSS Statistics for Windows, version 25 (IBM, Armonk, NY, USA) [40] were used as data processing and analysis programs. The ANOVA test was used to compare groups regarding related variables (anxiety, difficulties, coping). In particular, the Bonferroni Procedure was used for reducing the risk of detecting false-positive results due to multiple analyses. Finally, Pearson’s correlation coefficient was used for testing the correlation hypotheses in the case of subscales that measure anxiety, difficulties, and coping strategies. All statistical tests with a value of *p* < 0.05 were considered statistically significant. Variables whose distribution was not parametric were logarithmic according to the procedure proposed by Andy Field (Supplementary Materials Table S1 and S2) [41].

### 3.3. Testing the Study Hypothesis

For testing the first hypothesis of the study, the ANOVA test was used, which indicated that there were no significant differences regarding the scores of anxiety scales and difficulties between the participants who face different infertility time intervals (*p* > 0.05). Thus, increased duration of infertility in women did not seem to be associated with higher levels of anxiety and perceived difficulties.

The results of the unifactorial variance analysis, for testing the second hypothesis, showed that there were significant differences regarding the scores of the state anxiety scales [F(2, 237) = 4.76, *p* = 0.009] and the difficulty subscale “Impact on self and spouse” [F(2, 237) = 3.53, *p* = 0.031] among participants who resorted to repeated fertilization procedures compared to those who had not yet started treatment or were at the beginning of treatment (Table 2).

Post-hoc analyzes with Bonferroni-type adjustment confirmed that regarding state anxiety, there were statistically significant differences between participants who had undergone repeated treatment procedures compared to those who had not undergone any treatment thus far (difference between means −4.81, *p* = 0.015, confidence interval of the differences between the 95% level averages −8.93–−0.69).

However, contrary to the expectations of our second hypothesis, statistical analyzes showed that women who were at the beginning of treatment obtained higher scores on the anxiety and difficulty scales compared to participants who resorted to repeated fertilization procedures.

For testing the third, fourth, and fifth hypotheses of the study, we used the Pearson Correlation standard test (Table 3, Table 4 and Table 5). Therefore, it is important to understand that there was a significant positive correlation between the perception of infertility difficulties and the choice of coping strategies, regardless of the nature of the coping strategy (adaptive or maladaptive), except for the humor and acceptance that correlated negatively with the difficulties.

Between difficulties of infertile women and state anxiety, significant positive associations were observed.

Regarding the relationship between state anxiety and coping, there were significant positive associations between maladaptive coping strategies (self-distraction, substance use, self-blame) and state anxiety, while adaptive strategies (emotional support, positive reframing, humor, acceptance) were negatively associated with state anxiety. However, venting was positively associated with state anxiety.

For testing the sixth hypothesis of the study, an ANOVA test was used. The results indicated that there were significant differences regarding the scores of the state anxiety [F(3, 236) = 2.67, *p* = 0.048] and the coping subscales “Venting” [F(3,236) = 4.43, *p* = 0.005] and “Self-blame” [F(3, 236) = 4.46, *p* = 0.005] among participants who had different causes regarding the diagnosis of infertility (Table 6).

Post-hoc analysis with Bonferroni correction did not identify significant differences in the state-anxiety score between participants who had different causes of infertility. In contrast, significant differences were noted regarding the scores on the coping subscales “Venting” and “Self-blame.”

Regarding the subscale “Venting,” there were differences between participants in which the cause was feminine compared to those who reported the cause of infertility as being both feminine and masculine (difference between means 0.04, *p* = 0.044, confidence interval of differences between mean levels 95% 0.0007–0.0835), as well as between those in which the cause was feminine compared to those in which the cause of infertility was unexplained (difference between means 0.04, *p* = 0.029, confidence interval of differences between means 95% level 0.0031–0.0901).

In addition, regarding the subscale “Self-blame,” there were differences between the participants in which the cause was feminine compared to those who stated that the problem of infertility was caused by the partner (male cause) (difference between means 0.01, *p* = 0.010, confidence interval of differences between mean levels 95% 0.0190–0.2120).

## 4. Discussion

Infertility is a global public health issue. This diagnosis has a powerful impact on women’s lives, mainly from an emotional and social perspective.

The present study aims to highlight how women adapt to infertility difficulties and also explores the relationship between the difficulties and anxiety of infertile women.

Following the statistical analysis of the data, the following conclusions can be drawn. One of the possible explanations for which the first hypothesis has not been confirmed is that anxiety has clinical values regardless of the moment of discovery. Another explanation could be the factors that support anxiety and difficulties regardless of the duration of infertility: Family and social pressure [42], low self-esteem [43].

Contrary to expectations, women who have not yet begun or are beginning fertilization have higher scores on the anxiety scale, similar results being found in the literature [12]. These high levels of anxiety are maintained regardless of age, marital status, educational status, and duration of infertility. Equally, difficulties arise in terms of the impact on oneself and the spouse, which are an added source of stress. These difficulties can lead to a decrease in self-esteem, perception of life situation, problems in the marital relationship, as well as differences of opinion.

Looking at the correlations between the difficulties scale and coping strategies, we note the choice of dysfunctional coping methods, such as self-distraction, denial, substance use, behavioral disengagement, self-blame. This can be caused by the new mental state of infertile women and the lack of medical support. The diagnosis is difficult to accept, and the health system does not always cover the necessary treatment. Except for the humor and acceptance that correlates negatively with the difficulties, this draws attention to the fact that infertile women live this experience at very high levels of anxiety, using quite a few adaptive coping mechanisms. These results highlight the need to investigate ways to reduce anxiety and optimizing adaptive coping strategies throughout the IVF cycle [11], and to promote mental health and wellbeing of infertile women.

Difficulties were expected to correlate positively with anxiety due to the multiple pressures felt by infertile women [20]. A recent meta-analysis based on studies from the US and Europe also showed that a blocked parenthood goal can lead to greater anxiety yet not necessarily to disengagement from the goal, and even when women disengage, it does not reduce their anxiety [26]. Furthermore, Vaughan et al. [44] consider that despite the unprecedented global pandemic of COVID-19, causing economic and societal uncertainty, the problem of infertility remains significant and is a comparable stressor to the pandemic itself.

What attracts attention in our study results is the relationship between anxiety and coping, more specifically, the positive relationship between anxiety and venting. Adaptive strategies such as venting were expected to be negatively associated with anxiety. However, it seems that this aspect increases anxiety. One explanation, which is worth exploring in future studies, could be the fact that when women talk about their infertility difficulties, they cause them discomfort and deepen their existing suffering.

Analyzing the sixth hypothesis of the study, anxiety does not lose its meaning or level, regardless of the cause of infertility. Explicit studies are needed for each cause on different populations of infertile women. Regarding coping strategies, venting and self-blame occur predominantly in women who know that the cause of infertility is female-related. Venting that refers to emotional expression (which occurs more often in women whose infertility has a female cause, according to descriptive statistics), is a coping strategy used by infertile women to manage their life context. The levels of self-blame draw attention to the need to reduce this inadequate strategy and to investigate the phenomenon in the literature.

The present study has numerous strengths that should be considered, including:

(a) The examination of the anxiety symptoms and difficulties according to certain demographic and clinical characteristics, such as level of education, marital status, duration, type, and cause of infertility;
(b) The evaluation was carried out in several cities of Romania, which allows generalization of the results, unlike other studies that include participants from a single fertilization clinic.

The clinical implications of the study are obvious and should arouse the interest of specialists in order to improve the treatment of infertile women. At the same time, these implications highlight the need for a better understanding of infertility from a psychological point of view.

Future research could take into account other psychological variables (related to both partners), such as their self-esteem and quality of life, since a low quality of life is commonly associated with infertility or with different diseases like endometriosis [45], but also their relationship with health care providers from specialized fertilization clinics.

## 5. Conclusions

In conclusion, the results of the present study expand the literature, highlighting specific associations between anxiety, difficulties, and coping. It seems that women who are at the beginning of treatment are more emotionally affected than those who have gone through several treatment procedures. The difficulties perceived by women are closely related to their anxiety and the choice of dysfunctional coping methods. Regarding coping strategies, the emotions of venting and self-blame are associated with female infertility.

The current study has a limitation that deserves attention: The cross-sectional design. Therefore, no causal inferences can be made about the connections between variables. A methodological limitation is that the STAI-Form Y and Brief COPE are also used for general measurements and do not evaluate in the specific context of infertility.

Future studies examining the longitudinal association between different psychological variables will serve in clarifying whether the connections are truthfully relevant to the problem or not.

A strength point is that the mental health of infertile women could be improved through psychoeducation and therapy by learning how to use different coping strategies.

## Figures and Tables

**Table 1:** Demographic and fertility characteristics of the infertile women.

Characteristics	*N* = 240 Women
Age, years (mean)	33 (SD ^1^ = 4.8)
Education	
Elementary school	2.9%
High school	12.6%
Post-secondary school	10.8%
Without bachelor’s degree	2.9%
Bachelor’s degree	43.8%
Postgraduate degree	25%
Marital status	
Married	88.8%
Live with a partner	10.4%
Preferred not to answer this question	0.8%
Duration of infertility	
Less than 2 years	17.9%
2–5 years	43.3%
More than 5 years	38.8%
Type of infertility	
Primary	65.8%
Secondary	34.2%
Cause of infertility	
Female	36.7%
Male	17.1%
Both	25%
Idiopathic/unexplained	21.3%
FIV treatment	
Not yet	42.1%
One treatment	16.7%
Multiple treatment	41.3%

^1^ SD = Standard deviation.

**Table 2:** Analysis of the variance of anxiety and difficulties scores of the women in the study for different levels of treatment.

		Sum of Squares	Df ^2^	Mean Square	F	Sig. ^1^
State anxiety	Between Groups	0.113	2	0.057	4.698	0.010
	Within Groups	2.859	237	0.012		
	Total	2.972	239			
Difficulties-Uncertainty and lack of control	Between Groups	0.038	2	0.019	0.839	0.433
	Within Groups	5.344	237	0.023		
	Total	5.382	239			
Difficulties-Family and social pressures	Between Groups	0.077	2	0.038	1.589	0.206
	Within Groups	5.710	237	0.024		
	Total	5.786	239			
Difficulties-Impact on self and spouse	Between Groups	0.150	2	0.075	3.537	0.031
	Within Groups	5.010	237	0.021		
	Total	5.160	239			
Difficulties-Procedures related to treatment	Between Groups	0.080	2	0.040	1.368	0.257
	Within Groups	6.915	237	0.029		
	Total	6.995	239			
Difficulties-overall score	Between Groups	0.050	2	0.025	1.873	0.156
	Within Groups	3.160	237	0.013		
	Total	3.210	239			

^1^ Sig = Statistical significance; ^2^ Df = degrees of freedom.

**Table 3:** Correlations between coping strategies and difficulties of infertile women.

	Difficulties—Uncertainty and Lack of Control	Difficulties—Family and Social Pressures	Difficulties—Impact on Self and Spouse	Difficulties—Procedures Related to Treatment	Difficulties—Overall Score
Self-distraction	00.195 **	0.196 **	0.140 *	0.068	0.190 **
Active coping	0.189 **	0.160 *	0.075	0.046	0.173 **
Denial	0.125	0.251 **	0.193 **	0.155 *	0.207 **
Substance use	0.170 **	0.084	0.125	0.068	0.129 *
Emotional support	−0.114	−0.004	−0.112	−0.018	−0.085
Use of informational support	0.047	0.080	−0.006	0.139 *	0.071
Behavioral disengagement	0.168 **	0.183 **	0.216 **	0.157 *	0.221 **
Venting	0.185 **	0.070	0.129 *	0.109	0.164 *
Positive reframing	−0.064	0.046	−0.019	−0.019	−0.033
Planning	0.159 *	0.186 **	0.097	−0.033	0.152 *
Humor	0.041	−0.076	−0.086	−0.136 *	−0.059
Acceptance	−0.150 *	−0.158 *	−0.177 **	0.011	−0.165 *
Religion	−0.001	0.151 *	0.046	0.162 *	0.090
Self−blame	0.442 **	0.498 **	0.513 **	0.199 **	0.515 **
Emotion based coping	0.305 **	0.393 **	0.319 **	0.213 **	0.372 **
Problem based coping	0.106	0.107	−0.016	0.078	0.094

* Correlation is significant at the 0.05 level (2-tailed)./** Correlation is significant at the 0.01 level (2-tailed).

**Table 4:** Correlations between anxiety and difficulties of infertile women.

	State Anxiety
Difficulties-Uncertainty and lack of control	0.516 **
Difficulties-Family and social pressures	0.412 **
Difficulties-Impact on self and spouse	0.434 **
Difficulties-Procedures related to treatment	0.174 **
Difficulties-overall score	0.493 **

** Correlation is significant at the 0.01 level (2-tailed).

**Table 5:** Correlations between coping strategies and anxiety of infertile women.

	State Anxiety
Self-distraction	0.157 *
Active coping	0.016
Denial	0.076
Substance use	0.164 *
Emotional support	−0.269 **
Use of informational support	−0.067
Behavioral disengagement	0.030
Venting	0.136 *
Positive reframing	−0.215 **
Planning	0.103
Humor	−0.190 **
Acceptance	−0.313 **
Religion	−0.071
Self-blame	0.521 **
Emotion based coping	0.138 *
Problem based coping	−0.119

* Correlation is significant at the 0.05 level (2-tailed)./** Correlation is significant at the 0.01 level (2-tailed).

**Table 6:** Analysis of the variance of anxiety, difficulties, and coping scores of the women in the study according to the cause of infertility.

		Sum of Squares	Df ^2^	Mean Square	F	Sig. ^1^
State anxiety	Between Groups	0.100	3	0.033	2.674	0.048
	Within Groups	2.873	236	0.012		
	Total	2.972	239			
Difficulties—Uncertainty and lack of control	Between Groups	0.076	3	0.025	1.129	0.338
	Within Groups	5.306	236	0.022		
	Total	5.382	239			
Difficulties—Family and social pressures	Between Groups	0.044	3	0.015	0.604	0.613
	Within Groups	5.742	236	0.024		
	Total	5.786	239			
Difficulties—Impact on self and spouse	Between Groups	0.137	3	0.046	2.139	0.096
	Within Groups	5.023	236	0.021		
	Total	5.160	239			
Difficulties—Procedures related to treatment	Between Groups	0.054	3	0.018	0.611	0.608
	Within Groups	6.941	236	0.029		
	Total	6.995	239			
Difficulties—Overall score	Between Groups	0.054	3	0.018	1.339	0.262
	Within Groups	3.156	236	0.013		
	Total	3.210	239			
Self-distraction	Between Groups	0.010	3	0.003	0.492	0.688
	Within Groups	1.650	236	0.007		
	Total	1.660	239			
Active coping	Between Groups	0.023	3	0.008	0.767	0.513
	Within Groups	2.383	236	0.010		
	Total	2.406	239			
Denial	Between Groups	0.182	3	0.061	1.406	0.242
	Within Groups	10.167	236	0.043		
	Total	10.349	239			
Substance use	Between Groups	0.082	3	0.027	1.651	0.178
	Within Groups	3.922	236	0.017		
	Total	4.005	239			
Emotional support	Between Groups	0.021	3	0.007	0.689	0.559
	Within Groups	2.398	236	0.010		
	Total	2.419	239			
Use of informational support	Between Groups	0.012	3	0.004	0.194	0.900
	Within Groups	4.731	236	0.020		
	Total	4.743	239			
Behavioral disengagement	Between Groups	0.011	3	0.004	0.164	0.921
	Within Groups	5.396	236	0.023		
	Total	5.407	239			
Venting	Between Groups	0.115	3	0.038	4.430	0.005
	Within Groups	2.039	236	0.009		
	Total	2.153	239			
Positive reframing	Between Groups	0.046	3	0.015	1.598	0.191
	Within Groups	2.241	236	0.009		
	Total	2.287	239			
Planning	Between Groups	0.005	3	0.002	0.403	0.751
	Within Groups	1.025	236	0.004		
	Total	1.031	239			
Humor	Between Groups	0.079	3	0.026	2.057	0.107
	Within Groups	3.037	236	0.013		
	Total	3.116	239			
Acceptance	Between Groups	0.021	3	0.007	0.814	0.487
	Within Groups	2.030	236	0.009		
	Total	2.051	239			
Religion	Between Groups	0.032	3	0.011	0.364	0.779
	Within Groups	6.958	236	0.029		
	Total	6.991	239			
Self-blame	Between Groups	0.493	3	0.164	4.466	0.005
	Within Groups	8.679	236	0.037		
	Total	9.172	239			
Emotion based coping	Between Groups	0.008	3	0.003	0.907	0.439
	Within Groups	0.671	236	0.003		
	Total	0.678	239			
Problem based coping	Between Groups	0.002	3	0.001	0.246	0.864
	Within Groups	0.682	236	0.003		
	Total	0.684	239			

^1^ Sig = Statistical significance; ^2^ Df = degrees of freedom.

## Data Availability

The datasets generated during and/or analyzed during the current study are available from the corresponding author on reasonable request.

## Supplementary Materials

The following are available online at https://www.mdpi.com/article/10.3390/healthcare9040466/s1, Table S1. Descriptive statistics of anxiety and difficulties scores of the women in the study for different levels of treatment; Table S2. Descriptive statistics of anxiety, difficulties and coping scores of the women in the study according to the cause of infertility.
