# Supplementary material for: Anxiety, Difficulties, and Coping of Infertile Women
Source: Healthcare (Basel). 2021 Apr 15;9(4):466. doi: 10.3390/healthcare9040466 (PMC8071148; doi:10.3390/healthcare9040466)
Supplement: Supplementary file 1 [file healthcare-09-00466-s001.zip › healthcare-1154665-SI.pdf]

healthcare

MDPI

Supplementary Materials:

Table S1. Descriptive statistics of anxiety and difficulties scores of the women in the study for different levels of treatment.

		N	Mean	SD[1]	Std. Error	95% Confidence Interval for Mean		Min.	Max.
						Lower Bound	Upper Bound		
State-anxiety	Repeated treatment	99	1.6754	.11166	.01122	1.6531	1.6976	1.34	1.85
	Only one treatment	40	1.6713	.12144	.01920	1.6324	1.7101	1.34	1.90
	No treatment	101	1.7181	.10304	.01025	1.6978	1.7385	1.41	1.89
	Total	240	1.6927	.11152	.00720	1.6785	1.7068	1.34	1.90
Trait-anxiety	Repeated treatment	99	1.7171	.08682	.00873	1.6997	1.7344	1.43	1.86
	Only one treatment	40	1.6960	.09730	.01538	1.6648	1.7271	1.41	1.86
	No treatment	101	1.7475	.06851	.00682	1.7339	1.7610	1.54	1.86
	Total	240	1.7263	.08353	.00539	1.7157	1.7370	1.41	1.86
Difficulties-Uncertainty and lack of control	Repeated treatment	99	1.2864	.16301	.01638	1.2539	1.3189	.78	1.48
	Only one treatment	40	1.2657	.12939	.02046	1.2243	1.3071	.90	1.45
	No treatment	101	1.3014	.14447	.01438	1.2729	1.3299	.78	1.48
	Total	240	1.2893	.15007	.00969	1.2702	1.3083	.78	1.48
Difficulties- Family and social pressures	Repeated treatment	99	1.2186	.16179	.01626	1.1863	1.2508	.70	1.40
	Only one treatment	40	1.2228	.16240	.02568	1.1708	1.2747	.70	1.40
	No treatment	101	1.2558	.14546	.01447	1.2271	1.2845	.70	1.40
	Total	240	1.2349	.15560	.01004	1.2152	1.2547	.70	1.40
Difficulties- Impact on self and spouse	Repeated treatment	99	1.2269	.16195	.01628	1.1946	1.2592	.78	1.48
	Only one treatment	40	1.2074	.13176	.02083	1.1653	1.2496	.90	1.45
	No treatment	101	1.2700	.13276	.01321	1.2438	1.2962	.78	1.48
	Total	240	1.2418	.14693	.00948	1.2231	1.2605	.78	1.48
Difficulties- Problems induced by treatment	Repeated treatment	99	.8132	.13815	.01388	.7857	.8408	.30	1.00
	Only one treatment	40	.8239	.14823	.02344	.7765	.8713	.30	1.00
	No treatment	101	.8209	.16675	.01659	.7880	.8538	.30	1.00
	Total	240	.8183	.15187	.00980	.7989	.8376	.30	1.00
Difficulties- Procedures related to treatment	Repeated treatment	99	.9321	.17245	.01733	.8977	.9665	.48	1.18
	Only one treatment	40	.9642	.13109	.02073	.9223	1.0062	.60	1.18
	No treatment	101	.9707	.18250	.01816	.9347	1.0067	.48	1.18
	Total	240	.9537	.17108	.01104	.9319	.9754	.48	1.18

Difficulties- overall score	Repeated treatment	99	1.8418	.12134	.01219	1.8176	1.8660	1.43	2.04
	Only one treatment	40	1.8369	.09702	.01534	1.8059	1.8679	1.60	2.03
	No treatment	101	1.8694	.11619	.01156	1.8465	1.8924	1.40	2.03
	Total	240	1.8526	.11589	.00748	1.8379	1.8674	1.40	2.04

Table S2. Descriptive statistics of anxiety, difficulties and coping scores of the women in the study according to the cause of infertility.

		N	Mean	SD	Std. Error	95% Confidence Interval for Mean		Min.	Max.
						Lower Bound	Upper Bound		
State-anxiety	Female	88	1.7192	.10125	.01079	1.6978	1.7407	1.41	1.90
	Male	41	1.6812	.11045	.01725	1.6464	1.7161	1.38	1.86
	Both	60	1.6734	.10906	.01408	1.6453	1.7016	1.34	1.87
	Unexplained	51	1.6787	.12578	.01761	1.6433	1.7140	1.34	1.89
	Total	240	1.6927	.11152	.00720	1.6785	1.7068	1.34	1.90
Trait-anxiety	Female	88	1.7378	.07495	.00799	1.7220	1.7537	1.41	1.86
	Male	41	1.7291	.08690	.01357	1.7017	1.7566	1.43	1.85
	Both	60	1.7188	.08038	.01038	1.6981	1.7396	1.48	1.85
	Unexplained	51	1.7131	.09710	.01360	1.6858	1.7404	1.41	1.85
	Total	240	1.7263	.08353	.00539	1.7157	1.7370	1.41	1.86
Difficulties-Uncertainty and lack of control	Female	88	1.3101	.13919	.01484	1.2806	1.3396	.78	1.48
	Male	41	1.2866	.13832	.02160	1.2430	1.3303	.78	1.46
	Both	60	1.2829	.15048	.01943	1.2440	1.3218	.78	1.48
	Unexplained	51	1.2630	.17432	.02441	1.2139	1.3120	.78	1.48
	Total	240	1.2893	.15007	.00969	1.2702	1.3083	.78	1.48
Difficulties- Family and social pressures	Female	88	1.2425	.15057	.01605	1.2106	1.2744	.70	1.40
	Male	41	1.2300	.16879	.02636	1.1767	1.2833	.70	1.40
	Both	60	1.2475	.13615	.01758	1.2123	1.2826	.85	1.40
	Unexplained	51	1.2112	.17530	.02455	1.1619	1.2605	.70	1.40
	Total	240	1.2349	.15560	.01004	1.2152	1.2547	.70	1.40
Difficulties- Impact on self and spouse	Female	88	1.2704	.13457	.01435	1.2419	1.2989	.78	1.48
	Male	41	1.2255	.14720	.02299	1.1790	1.2719	.78	1.43
	Both	60	1.2385	.12308	.01589	1.2067	1.2703	.90	1.41
	Unexplained	51	1.2095	.18369	.02572	1.1578	1.2612	.78	1.48
	Total	240	1.2418	.14693	.00948	1.2231	1.2605	.78	1.48
Difficulties- Problems induced by treatment	Female	88	.8228	.16577	.01767	.7877	.8579	.30	1.00
	Male	41	.8445	.13376	.02089	.8022	.8867	.30	1.00
	Both	60	.8300	.12036	.01554	.7989	.8611	.30	1.00
	Unexplained	51	.7755	.16852	.02360	.7281	.8229	.30	1.00
	Total	240	.8183	.15187	.00980	.7989	.8376	.30	1.00
Difficulties- Procedures related to treatment	Female	88	.9432	.18312	.01952	.9044	.9820	.48	1.18
	Male	41	.9577	.18447	.02881	.8994	1.0159	.48	1.18
	Both	60	.9777	.14709	.01899	.9397	1.0157	.48	1.18
	Unexplained	51	.9404	.16650	.02331	.8936	.9872	.48	1.18
	Total	240	.9537	.17108	.01104	.9319	.9754	.48	1.18
Difficulties- overall score	Female	88	1.8657	.11585	.01235	1.8411	1.8902	1.43	2.04
	Male	41	1.8523	.10119	.01580	1.8204	1.8842	1.54	2.01

	Both	60	1.8568	.09727	.01256	1.8317	1.8819	1.52	2.01
	Unexplained	51	1.8254	.14289	.02001	1.7853	1.8656	1.40	2.02
	Total	240	1.8526	.11589	.00748	1.8379	1.8674	1.40	2.04
Self-distraction	Female	88	.7913	.08040	.00857	.7742	.8083	.48	.90
	Male	41	.7965	.07214	.01127	.7737	.8193	.60	.90
	Both	60	.7780	.08933	.01153	.7549	.8011	.48	.90
	Unexplained	51	.7850	.09040	.01266	.7596	.8104	.48	.90
	Total	240	.7875	.08335	.00538	.7769	.7981	.48	.90
Active coping	Female	88	.8079	.11959	.01275	.7825	.8332	.30	.90
	Male	41	.8120	.07841	.01225	.7872	.8367	.60	.90
	Both	60	.8286	.08187	.01057	.8075	.8498	.60	.90
	Unexplained	51	.8019	.09971	.01396	.7739	.8299	.48	.90
	Total	240	.8125	.10033	.00648	.7997	.8253	.30	.90
Denial	Female	88	.6288	.21305	.02271	.5837	.6740	.30	.90
	Male	41	.5794	.22094	.03450	.5096	.6491	.30	.90
	Both	60	.5648	.20331	.02625	.5122	.6173	.30	.90
	Unexplained	51	.6187	.19116	.02677	.5649	.6724	.30	.90
	Total	240	.6022	.20809	.01343	.5757	.6287	.30	.90
Substance use	Female	88	.3626	.15076	.01607	.3307	.3946	.30	.90
	Male	41	.3231	.07937	.01240	.2980	.3481	.30	.60
	Both	60	.3315	.10732	.01385	.3038	.3592	.30	.78
	Unexplained	51	.3689	.14236	.01993	.3288	.4089	.30	.78
	Total	240	.3494	.12944	.00836	.3329	.3659	.30	.90
Emotional support	Female	88	.7547	.09629	.01026	.7343	.7751	.48	.90
	Male	41	.7672	.09204	.01437	.7381	.7962	.60	.90
	Both	60	.7769	.09415	.01215	.7526	.8012	.48	.90
	Unexplained	51	.7731	.12079	.01691	.7391	.8071	.48	.90
	Total	240	.7663	.10060	.00649	.7535	.7791	.48	.90
Use of informational support	Female	88	.7164	.14047	.01497	.6866	.7461	.30	.90
	Male	41	.7249	.14650	.02288	.6786	.7711	.30	.90
	Both	60	.7191	.14964	.01932	.6805	.7578	.30	.90
	Unexplained	51	.7346	.12924	.01810	.6982	.7709	.30	.90
	Total	240	.7224	.14088	.00909	.7045	.7403	.30	.90
Behavioral disengagement	Female	88	.4865	.15004	.01599	.4547	.5183	.30	.85
	Male	41	.4794	.15116	.02361	.4317	.5271	.30	.70
	Both	60	.4983	.14899	.01923	.4598	.5368	.30	.78
	Unexplained	51	.4822	.15581	.02182	.4384	.5261	.30	.85
	Total	240	.4873	.15041	.00971	.4682	.5065	.30	.85
Venting	Female	88	.7950	.09416	.01004	.7750	.8149	.60	.90
	Male	41	.7935	.08039	.01255	.7682	.8189	.60	.90
	Both	60	.7528	.08613	.01112	.7306	.7751	.48	.90
	Unexplained	51	.7483	.10687	.01496	.7183	.7784	.48	.90
	Total	240	.7743	.09492	.00613	.7622	.7863	.48	.90
Positive reframing	Female	88	.7264	.08735	.00931	.7079	.7449	.48	.90
	Male	41	.7589	.09215	.01439	.7298	.7880	.48	.85
	Both	60	.7298	.10195	.01316	.7034	.7561	.30	.90
	Unexplained	51	.7158	.11176	.01565	.6844	.7473	.30	.90
	Total	240	.7305	.09782	.00631	.7181	.7430	.30	.90
Planning	Female	88	.8364	.06391	.00681	.8229	.8499	.60	.90
	Male	41	.8228	.06944	.01084	.8009	.8447	.70	.90
	Both	60	.8332	.06520	.00842	.8163	.8500	.70	.90

	Unexplained	51	.8316	.06730	.00942	.8127	.8505	.70	.90
	Total	240	.8323	.06567	.00424	.8239	.8406	.60	.90
Humor	Female	88	.6876	.10548	.01124	.6653	.7100	.30	.90
	Male	41	.7318	.10222	.01596	.6995	.7640	.48	.90
	Both	60	.6994	.11582	.01495	.6695	.7294	.30	.90
	Unexplained	51	.6762	.13111	.01836	.6393	.7131	.30	.90
	Total	240	.6957	.11419	.00737	.6812	.7102	.30	.90
Acceptance	Female	88	.7762	.09307	.00992	.7565	.7959	.48	.90
	Male	41	.7950	.07129	.01113	.7725	.8175	.60	.90
	Both	60	.7857	.08596	.01110	.7635	.8079	.48	.90
	Unexplained	51	.7670	.11293	.01581	.7353	.7988	.30	.90
	Total	240	.7798	.09265	.00598	.7681	.7916	.30	.90
Religion	Female	88	.7041	.18277	.01948	.6654	.7429	.30	.90
	Male	41	.7325	.14427	.02253	.6870	.7780	.48	.90
	Both	60	.7220	.17969	.02320	.6755	.7684	.30	.90
	Unexplained	51	.7287	.16216	.02271	.6831	.7743	.30	.90
	Total	240	.7187	.17102	.01104	.6969	.7404	.30	.90
Self-blame	Female	88	.7441	.17439	.01859	.7072	.7811	.30	.90
	Male	41	.6286	.20559	.03211	.5637	.6935	.30	.90
	Both	60	.6662	.20090	.02594	.6143	.7181	.30	.90
	Unexplained	51	.7296	.19807	.02774	.6739	.7853	.30	.90
	Total	240	.7019	.19590	.01265	.6769	.7268	.30	.90
Emotion based coping	Female	88	1.7026	.05431	.00579	1.6911	1.7141	1.56	1.83
	Male	41	1.6963	.05316	.00830	1.6795	1.7130	1.57	1.80
	Both	60	1.6879	.05201	.00671	1.6745	1.7014	1.49	1.80
	Unexplained	51	1.6977	.05315	.00744	1.6827	1.7126	1.59	1.80
	Total	240	1.6968	.05327	.00344	1.6900	1.7036	1.49	1.83
Problem based coping	Female	88	1.3962	.06021	.00642	1.3834	1.4089	1.23	1.49
	Male	41	1.3993	.04648	.00726	1.3846	1.4140	1.30	1.49
	Both	60	1.4030	.05216	.00673	1.3895	1.4165	1.23	1.48
	Unexplained	51	1.3956	.04886	.00684	1.3819	1.4094	1.28	1.48
	Total	240	1.3983	.05349	.00345	1.3915	1.4051	1.23	1.49
